# Acute and Chronic Effects of Green Tea Kombucha With and Without Grape Skin Extract on Immune and Oxidative Stress Markers in Beginner Runners: A Randomized Controlled Trial

**DOI:** 10.1111/1750-3841.71505

**Published:** 2026-09-26

**Authors:** Dandara Baia Bonifácio, Rayanne Santos de Paulo, Pedro Henrique Viana Mendes, Udielle Vermelho Lacerda, Rodrigo Rezende Cardoso, Helton de Sá Souza, Frederico Augusto Ribeiro de Barros, Josefina Bressan

**Affiliations:** ^1^ Department of Nutrition and Health Universidade Federal de Viçosa Viçosa Brazil; ^2^ Department of Physical Education Universidade Federal de Viçosa Viçosa Brazil; ^3^ Department of Food and Technology Universidade Federal de Viçosa Viçosa Brazil

**Keywords:** antioxidants, catechins, exercise, fermentation

## Abstract

Green tea kombucha is an antioxidant‐rich beverage with promising health benefits. However, evidence regarding its effects in physically active individuals remains limited. This study investigated the acute and chronic effects of green tea kombucha, with or without grape skin extract, on immune and oxidative stress markers in beginner runners. This 12‐week randomized clinical trial included 90 healthy adults (18–45 years) allocated to three groups: control (CT‐G), green tea kombucha (GT‐G), and green tea kombucha with grape skin extract (GTG‐G). Participants performed supervised running training three times weekly following a polarized periodization model. Kombucha intake was not associated with significant changes in the primary outcome, ferric reducing antioxidant power (FRAP), nor in acute immune or oxidative responses to exercise (*p* > 0.05). However, during the chronic period, the GT‐G showed increased neutrophils (*p* = 0.026) and reduced lymphocytes (*p* = 0.023) compared to the other groups. Fasting catalase (CAT) activity differed between groups GT‐G and CT‐G (*p* = 0.037), despite no significant group x time interaction (*p* = 0.157). A group × time interaction was detected for post‐exercise malondialdehyde concentrations (*p* = 0.033), with no significant interaction in the post hoc test. Sensitivity analyses including all randomized participants confirmed the primary observations; however, false discovery rate (FDR) adjustments indicated these findings are exploratory. No additional effects were detected in GTG‐G. In conclusion, 12 weeks of green tea kombucha intake was associated with nominally significant changes in immune and oxidative markers during chronic running training, and further studies are needed to clarify these findings.

## 1 Introduction

Kombucha is a fermented beverage obtained from *Camellia sinensis* tea, sugar, and a symbiotic consortium of bacteria and yeasts known as SCOBY (Symbiotic Culture of Bacteria and Yeast) (Andrade et al. 2025; Onsun et al. 2025). The fermentation process yields an acidic and mildly carbonated beverage rich in bioactive compounds, including organic acids, vitamins, minerals, phenolic compounds, and microorganisms (Costa et al. 2023; Fraiz et al. 2024). These components have been associated with antioxidant and anti‐inflammatory properties, suggesting potential benefits for metabolic and immune health (Bonifácio et al. 2025; A. Chen et al. 2025).

In recent years, the search for new fermentable matrices has expanded kombucha diversity, aiming to achieve different sensory profiles and generate bioactive compounds with antioxidant potential (Khazi et al. 2024). Among these alternatives, grape skin stands out as it is rich in polyphenols, including anthocyanins (Capozzi et al. 2024; Elejalde et al. 2025). Evidence from a previous systematic review and meta‐analysis showed that both acute and chronic grape‐derived polyphenol intake can increase plasma antioxidant capacity in physically active individuals (Bonifácio et al. 2023). However, to date, no studies have specifically evaluated the potential synergistic effects of combining kombucha fermentation with grape skin‐derived bioactive compounds, particularly under clinically controlled experimental conditions.

Furthermore, the effects of kombucha consumption in the context of physical exercise have not yet been explored. Physical exercise, especially aerobic activities such as running, imposes significant physiological stress, leading to marked alterations in immune function and redox balance (Suzuki et al. 2025). The first line of defense of the immune system, composed of Natural Killer (NK) cells and phagocytes such as neutrophils, monocytes, and macrophages, exhibits high sensitivity to stimuli generated during an acute session of aerobic exercise (Quintana‐Mendias et al. 2023; Sever et al. 2025). Following vigorous efforts, an increase in cytotoxicity is observed, mainly due to the greater recruitment of NK cells and CD8^+^ T lymphocytes into the circulation (Nieman and Wentz, 2019). This activation requires immune cells to readjust their metabolism to ensure sufficient energy availability for expansion and the performance of effector functions. The redistribution of these cell populations to peripheral tissues may temporarily reduce immune efficiency (Campbell and Turner, 2018). After exercise cessation, leukocyte counts return to basal levels in a subset‐dependent manner. It is frequently followed by a marked decrease in the number of circulating cells, characterizing transient lymphopenia (Meyer‐Lindemann et al. 2023).

The increase in energy demand imposed by exercise also intensifies the generation of reactive oxygen and nitrogen species (RONS) (García‐Giménez et al. 2024). Several cellular structures contribute to this production, including mitochondria, phospholipase A2, and NADPH oxidases, particularly NOX2 and NOX4, distributed across the sarcolemma, sarcoplasmic reticulum, T‐tubules, and mitochondrial matrix (Powers et al. 2020). In parallel, exercise may increase body temperature and the concentrations of catecholamines and lactate, conditions that favor the conversion of the superoxide radical (O_2_•–) into the hydroxyl radical (•OH), thereby contributing to redox imbalance (García‐Giménez et al. 2024). In addition, repeated contractions increase nitric oxide production in muscle tissue, providing the necessary substrate for the formation of nitrogen‐derived reactive species (Powers et al. 2020).

This exercise‐related increase in redox stress may also be influenced by dietary bioactive compounds that act during training. Green tea catechins and other phenolic compounds can contribute to antioxidant protection through both direct interactions with reactive species and the activation of endogenous defense systems, including nuclear factor erythroid 2–related factor 2 (Nrf2)‐related pathways involved in the regulation of antioxidant enzymes (Ayubi et al. 2026; García‐Rodríguez et al. 2025). During repeated exercise, these mechanisms may help control excessive oxidative reactions and support redox balance, which may also influence immune responses (Meng and Su, 2024).

Accordingly, this study investigated whether kombucha supplementation could modulate acute and chronic immune and redox responses to exercise. Given the critical role of redox homeostasis in exercise physiology (Meng and Su,  2024), we hypothesized that kombucha supplementation, with or without grape skin extract, could modulate RONS dynamics and immune cell distribution in response to both acute post‐exercise and chronic running training.

## 2 Methods

### 2.1 Study Overview

This randomized, open‐label clinical trial included three parallel experimental groups: the control group (CT‐G), the green tea kombucha group (GT‐G), and the green tea kombucha with grape skin extract group (GTG‐G). The experimental protocol complies with international ethical guidelines, such as the Declaration of Helsinki. This study was approved by the Ethics Committee for Research with Human Beings (CAAE: 84876824.9.0000.5153) and prospectively registered in the Brazilian Registry of Clinical Trials (ReBEC; RBR‐6hxvph7; UTN U1111‐1321‐2251). This study began in 2025, and the trial registration record is available at https://ensaiosclinicos.gov.br/rg/RBR‐6hxvph7. All participants who agreed to take part signed the informed consent form.

The primary outcome was the change in plasma total antioxidant capacity after 12 weeks of intervention. No changes were made to the prespecified primary and secondary outcomes after trial registration. In this study, acute effects were defined as changes in outcomes assessed immediately before and after a single exercise session, whereas chronic effects referred to changes observed following the 12‐week intervention period, reflecting adaptations to repeated exercise and kombucha consumption.

### 2.2 Participants and Randomization

The sample size was calculated using G*Power version 3.1. The primary outcome considered was total plasma antioxidant capacity, based on the significant improvement observed in a randomized clinical trial investigating the effects of whole grape juice consumption on athletes (Goulart et al. 2020). An effect size of 0.80, a significance level (*α*) of 0.05, and a statistical power of 80% were assumed. Based on these parameters, the calculation indicated a minimum of 21 participants per group. Allowing for a 30% loss during the 12‐week intervention, the target sample size increased to 30 participants per group, for a total of 90 participants.

This study included adult men and women aged 18–45 years, with body mass index (BMI) between 18.5 and 24.9 kg/m^2^ and a level of physical activity classified as low to moderate according to the International Physical Activity Questionnaire—Short Version (IPAQ). Individuals with chronic diseases or digestive, oral, hepatic, renal, cardiovascular, inflammatory, or neoplastic disorders were not included, as were those who had used anti‐inflammatory drugs, corticosteroids, antibiotics, or antioxidant nutritional supplements in the last 3 months; alcohol consumers in quantities exceeding 21 units per week (men) or 14 units (women); pregnant, lactating, or menopausal women; smokers or drug users; individuals undergoing nutritional monitoring for muscle gain or weight loss; those with active or recurrent musculoskeletal injuries; those who habitually consume kombucha, other fermented foods, or green tea; or those with an aversion to kombucha.

Participants were allocated in a 1:1 ratio using the MinimPy software to one of three intervention groups: CT‐G, GT‐G, or GTG‐G. Randomization used the minimization method, as described by Abramson (2011), with sex, age, BMI, and physical activity level (measured by the IPAQ) as prognostic factors.

Study researchers enrolled participants and entered baseline information into the software, which generated group allocation according to the predefined minimization criteria. As this was an open‐label clinical trial, participants and study researchers were not blinded to treatment allocation. We did not use a placebo control because the characteristic sensory and physicochemical properties of kombucha, particularly its taste and acidity, are readily detectable, making adequate blinding difficult. Only laboratory personnel responsible for the biochemical analyses were blinded to group allocation and were unaware of the treatment assignments during the respective analyses.

### 2.3 Kombucha Production and Characterization

Kombucha was prepared from green tea leaves (*Camellia sinensis*) grown in Brazil, following the methodology described by Fraiz et al. (2024). The time–temperature combination was 70°C for 1 min. Tea from a previous batch of kombucha was added to the preparation, ensuring the initial pH was between 4.4 and 4.2 to prevent contamination and the proliferation of undesirable microorganisms. Fermentation took place in 20 L containers at 25°C for 5 days, with a SCOBY at a concentration of 30 g/L obtained from a certified company (Enziquímica Produtos Químicos Ltda, Gravataí, Brazil). For the GTG‐G kombuchas, an aqueous extract of BRS Vitória grape skin, purchased from the local market, was added at 15% by volume. To achieve this, the grapes were sanitized with a 100 ppm sodium hypochlorite solution for 10 min, rinsed, and the skins were manually separated from the pulp. The aqueous extract consisted of 1 part skin to 10 parts filtered water (1:10), processed in an industrial blender for 4 min, and strained to remove residues.

After production, the beverage was bottled and stored under refrigeration. Participants received seven individual bottles per week and were instructed to consume one 200‐mL bottle daily. Thus, the weekly production and distribution schedule ensured that the beverage was consumed within 1 week after production under refrigerated storage.

Although we did not formally assess batch‐to‐batch variability, we maintained standardized production conditions to minimize variability across weekly production cycles. Previous studies from our research group provide complementary information regarding green tea kombucha produced under similar standardized conditions, including detailed phenolic profiling and the characterization of physicochemical and microbiological parameters during refrigerated storage (Lacerda et al. 2025a, 2025b). These previous studies demonstrated the stability of key physicochemical parameters, including pH, total acidity, acetic acid concentration, ethanol content, and total phenolic content, while changes in residual sugars and microbial populations were monitored during storage.

Additionally, Table 1 presents the characterization of green tea kombucha with and without grape skin extract. A 200‐mL serving of GT‐G contained 4.45 g of sucrose, 2.51 g of fructose, 2.30 g of glucose, and 0.42 g of acetic acid, whereas the same serving size of GTG‐G contained 4.71 g of sucrose, 1.64 g of fructose, 1.10 g of glucose, and 0.35 g of acetic acid.

**TABLE 1:** Chemical and microbiological composition of kombuchas.

Components	GT‐G	GTG‐G
Sucrose (g/L)	22.24	23.56
Fructose (g/L)	12.57	8.21
Glucose (g/L)	11.49	5.49
Acetic acid (g/L)	2.12	1.73
Ethanol (g/L)	0.00032	0.00022
pH	3.41 (0.09)	3.65 (0.03)
Total acidity (w/v)	0.20 (0.02)	0.18 (0.03)
Lactic acid bacteria (CFU/mL)	1.98 × 10^7^	5.72 × 10^6^
Acetic acid bacteria (CFU/mL)	1.07 × 10^7^	5.76 × 10^6^
Yeast (CFU/mL)	1.57 × 10^7^	5.78 × 10^6^
Phenolic compounds (mg GAE/mL)	0.72 (0.02)	0.94 (0.03)

*Note*: Values are presented as means of three samples from different batches.

Abbreviation: GAE, gallic acid equivalent.

*Source*: Adapted from Fraiz et al. (2024).

### 2.4 Study Procedures

The study was publicized through the university's internal channels, posters, and social media. Those who expressed interest completed an online pre‐screening questionnaire. Individuals who met the preliminary inclusion criteria attended the Energy Metabolism and Body Composition Laboratory of the Department of Nutrition and Health (LAMECC/DNS‐UFV) for in‐person screening. During this stage, all inclusion criteria were evaluated. Sociodemographic, clinical, and lifestyle characteristics were also assessed using standardized questionnaires. A booklet with detailed instructions regarding blood collection, water consumption, and food intake on the previous day was also provided.

**FIGURE 1:**
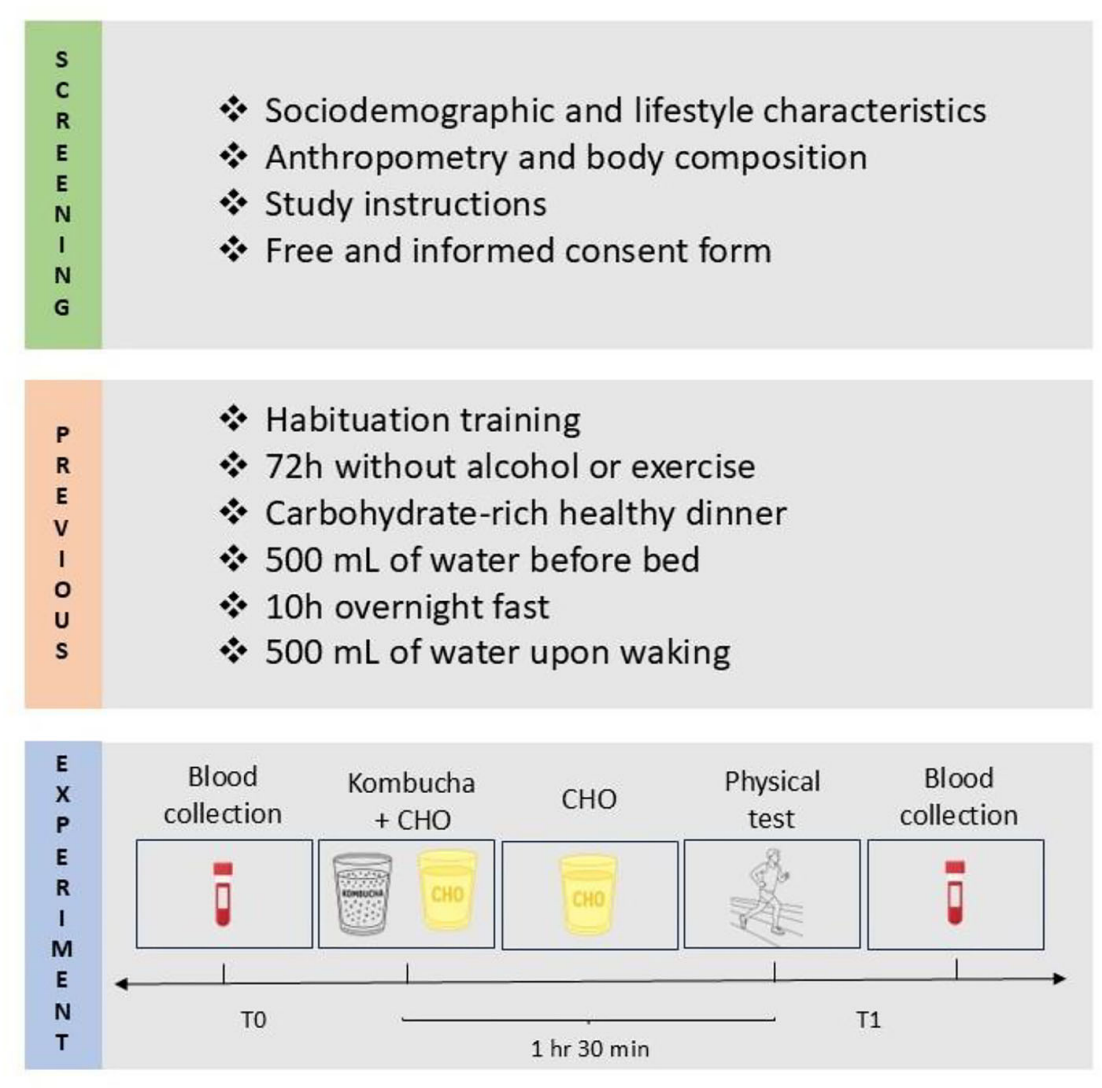
Flowchart of data collection for the clinical study.

During screening, body weight and muscle mass were measured using a digital scale equipped with a tetrapolar bioelectrical impedance system (InBody, model Y230). Height was measured using a vertical stadiometer with a maximum height of 2.2 m and a precision of 0.5 cm. Based on these measurements, the BMI was calculated. All measurements were taken after participants fasted for 3–4 h, were wearing light clothing, and were not wearing metallic accessories (Figure 1).

Before the experimental day, all participants attended a habituation training session on the athletics track. Participants abstained from alcohol and strenuous exercise for 72 h before the experimental day. The night before, they were instructed to consume healthy, carbohydrate‐rich meals and to avoid fatty, ultra‐processed foods and fast food. In addition, they ingested 500 mL of water before bedtime and the same amount on the morning of data collection, after a 10‐h overnight fast. For female participants, baseline and post‐intervention assessments were scheduled outside the menstrual bleeding period to minimize potential variability associated with menstruation.

On the day of the experiment, participants arrived at the Psychobiology and Exercise Laboratory (LAPSE) and had their blood drawn while fasting. They were instructed to consume 200 mL of kombucha (GT‐G and GTG‐G) or water (CT‐G), along with 250 mL of a carbohydrate solution (14.5 g), within 10 min. The carbohydrate solution was designed to enhance the absorption of kombucha polyphenols (Aatif, 2023). After consuming the beverages, participants remained seated for 1 h and 30 min until the next step. The postprandial time interval was selected based on evidence indicating that, in healthy individuals, catechins and their metabolites, which are the main bioactive compounds in kombucha, reach peak plasma levels between 1 and 2 h after ingestion (Barnett et al. 2015).

Note that 1 h and 30 min after ingesting the test beverages, all participants consumed an additional 250 mL of the carbohydrate solution (14.5 g) to prevent hypoglycemia during the physical tests. Following this, participants performed an incremental voluntary exhaustion test, after which another blood sample was collected. All tests were conducted at the same time each day to minimize potential interference from the circadian rhythm (Teo et al. 2011).

The incremental test to voluntary exhaustion was performed on an athletics track according to a pre‐established protocol (Amorim et al. 2025). Participants began the test with a 3‐min run at 8 km/h. The speed was increased by 1 km/h every 3 min until maximum voluntary exhaustion occurred. The speed at each stage was controlled by a researcher riding a bicycle equipped with a precision speedometer, positioned in front of each participant. To ensure proper monitoring of the test, 20 cones were placed along the track, spaced every 20 m. Maximum voluntary exhaustion was defined as the point at which the participant was more than 20 m behind the cyclist or when their subjective perception of effort indicated complete exhaustion.

### 2.5 Study Protocol and Discontinuation Criteria

Participants were instructed regarding their habitual physical activity levels and received standardized guidance from qualified staff to perform maintenance running sessions three times per week at the athletics track. All participants followed a polarized running model, characterized by a “U‐shaped” distribution of training intensity, with approximately 80% of total training time performed at low intensity (Z1–Z2), ∼15% at high intensity (Z4–Z5), and < 5% at moderate intensity (Z3) (Stöggl and Sperlich, 2015; Treff et al. 2019).

Training intensity zones were defined based on the peak running velocity achieved during the maximal incremental exercise test (Amorim et al. 2025). The peak velocity attained at the end of the test was used to establish five training intensity zones according to the percentages proposed by Seiler (2010): Z1 (50%–60%), Z2 (66%–80%), Z3 (81%–87%), Z4 (88%–93%), and Z5 (94%–100%). During the sessions, participants monitored the prescribed intensity using target lap times corresponding to each training zone on the athletics track. A qualified physical education professional continuously supervised all training sessions. Training volume and load were progressively adjusted throughout the 12‐week intervention according to the principles of undulating periodization, with a mean weekly increase of 1.57 km. The researchers monitored training‐load distribution using the Polarization Index (Treff et al. 2019), which remained between 2.0 and 2.2 throughout the intervention. The researchers monitored training adherence through attendance at the supervised running sessions, and participants who failed to complete at least 75% of the prescribed sessions were excluded from the study.

Participants were provided with records to document daily kombucha intake, which were collected at the end of the intervention. We also assessed adherence to kombucha consumption by tracking the return of the bottles provided to participants. At Week 6 adherence to the study protocol was assessed using a structured questionnaire that evaluated compliance with dietary and training recommendations, including avoidance of habitual consumption of antioxidant supplements and specific foods, such as fermented products and grape juice. The questionnaire also addressed dietary behavior, body weight changes, training adherence, muscle soreness, and included a subjective self‐rating of protocol adherence. Participants were excluded for poor adherence, including consumption of antioxidant supplements, corticosteroids, antibiotics, or anti‐inflammatory drugs, as well as failure to consume kombucha for three or more consecutive days. At the mid‐intervention assessment, participants also reported any adverse events or unintended effects experienced during the study.

### 2.6 Food Consumption

At the beginning and end of the study, dietary intake was evaluated using a food diary for 3 days and subsequently processed using the ERICA‐REC24h digital platform. Nutritional composition data were based on the Brazilian Institute of Geography and Statistics (IBGE) food composition database (IBGE 2011). All dietary variables were energy‐adjusted to 1000 kcal to account for differences in total energy intake.

### 2.7 Blood Sampling and Biochemical Analyses

During the initial and final experimental days, participants underwent fasting blood collection and post‐exercise blood collection. All samples were collected by a qualified professional using serology and EDTA tubes. All measurements were performed at the Clinical and Genomic Analysis Laboratory (LACEG) of UFV. The remaining materials were centrifuged at 3500 rpm for 10 min at 4°C to separate serum and plasma, which were stored at −80°C until analysis. For baseline characterization of participants, fasting blood glucose was measured in plasma using the UV kinetic method with the BS‐200 biochemical analyzer.

All biochemical analyses were performed in duplicate within the same analytical batch, with laboratory personnel blinded to treatment allocation. Post‐exercise blood samples were collected within 10 min of exercise completion.

#### 2.7.1 Immune Evaluation

Cell counts of leukocytes, neutrophils, eosinophils, lymphocytes, monocytes, and platelets were performed using a complete blood count (CBC) on an automated hematology analyzer (Scatter Laser). The neutrophil‐to‐lymphocyte ratio (NLR), platelet‐to‐lymphocyte ratio (PLR), and monocyte‐to‐lymphocyte ratio (MLR) were calculated as previously described (Cai et al. 2024). Briefly, each ratio was obtained by dividing the absolute count of the respective cell type (neutrophils, platelets, or monocytes) by the absolute lymphocyte count.

#### 2.7.2 Oxidative Stress Evaluation

Oxidative stress was assessed in plasma obtained from blood samples collected under fasting and post‐exercise conditions. Colorimetric enzymatic assays were conducted to determine catalase (CAT) activity, superoxide dismutase (SOD) activity, ferric reducing antioxidant power (FRAP), hydrogen peroxide (H_2_O_2_) levels, malondialdehyde (MDA) concentrations, protein carbonyl content, and nitrite levels. Protein carbonyl levels were assessed only in the post‐exercise condition, as this marker is more responsive to acute exercise‐induced oxidative stress, enhancing its ability to detect transient redox perturbations.

CAT activity was further assessed by monitoring the decomposition of hydrogen peroxide into water and oxygen, as proposed by Goth (1991). SOD activity was evaluated by its ability to catalyze the dismutation of the superoxide anion (O_2_^−^) into hydrogen peroxide, thereby inhibiting the auto‐oxidation rate of pyrogallol, following the method of Marklund and Marklund (1974). FRAP was determined according to Benzie and Strain (1996) by measuring the reduction of ferric (Fe^3^^+^) to ferrous (Fe^2^^+^) ions in the presence of electron‐donating antioxidants.

The H_2_O_2_ concentrations were determined by a spectrophotometric assay based on the horseradish peroxidase (HRP)‐catalyzed oxidation of o‐dianisidine (ODP), adapted from Möller and Ottolenghi (1966). Lipid peroxidation was estimated using the thiobarbituric acid reactive substances (TBARS) assay, with results expressed as MDA equivalents, as described by Buege and Aust (1978). Protein carbonyl content was determined by derivatization with 2,4‐dinitrophenylhydrazine (DNPH), resulting in the formation of stable dinitrophenylhydrazone derivatives, which were subsequently quantified spectrophotometrically, according to Levine et al. (1990). Nitrite levels were assessed using the Griess reaction, which measures nitrite as a stable end product, as described by Grisham et al. (1996).

### 2.8 Statistical Analyses

All statistical analyses were performed using Jamovi software (version 2.7.16). The level of significance was set at 5%. Baseline characteristics were analyzed using factorial analysis of variance (factorial ANOVA) for continuous variables and the chi‐square test for categorical variables.

The effects of the treatments were analyzed using linear mixed models with a repeated‐measures structure. Model assumptions were evaluated by visual inspection of the residuals. Each immune and oxidative stress marker was analyzed separately as a dependent variable. To assess both acute and chronic effects of the intervention, independent models were constructed: (1) a model for acute effects, considering pre‐ and post‐test incremental running time, and (2) a model for chronic effects, considering pre‐ and post‐intervention time (12 weeks).

In both models, group (CT‐G, GT‐G, and GTG‐G) and time (pre and post) were included as fixed effects, along with the group × time interaction. Models were estimated using restricted maximum likelihood (REML). When a significant interaction was observed, post hoc analyses were conducted using a Bonferroni correction for multiple comparisons. Additionally, Spearman correlation was performed in the post‐exercise period between markers of immunity and oxidative stress. All *p*‐values for the correlation were adjusted for multiple comparisons using the Benjamini–Hochberg false discovery rate (FDR) procedure.

The prespecified primary outcome was total plasma antioxidant capacity, assessed using the FRAP assay, based on the expected antioxidant effects of kombucha. Other oxidative stress and immune biomarkers were considered secondary outcomes. Correlation analyses were considered exploratory.

A sensitivity analysis was performed using all available observations from the randomized participants (*n* = 90) in the linear mixed‐effects models. We compared the results with those from the complete‐case analysis of participants who completed the chronic intervention period (*n* = 63). The sensitivity analysis was performed for outcomes showing nominally significant effects in the primary analysis, as well as for FRAP, the prespecified primary outcome.

To account for multiplicity across secondary outcomes, *p*‐values for the prespecified families of secondary outcome analyses were adjusted using the Benjamini–Hochberg FDR procedure, with an FDR threshold of 5%. Separate FDR adjustments were performed for the effects observed after the 12‐week intervention within four prespecified families of secondary outcomes: (1) immune markers assessed under fasting conditions, (2) immune markers assessed following exercise, (3) oxidative stress markers assessed under fasting conditions, and (4) oxidative stress markers assessed following exercise. FRAP was excluded from the FDR adjustment because it was prespecified as the primary outcome.

## 3 Results

### 3.1 Participants Characteristic

A total of 1120 individuals completed the online pre‐screening questionnaire. Of these, 1030 were excluded for the following reasons: failure to meet the inclusion criteria (*n* = 802), high levels of physical activity as assessed by the IPAQ (*n* = 151), and withdrawal from participation due to unavailability for training (*n* = 77).

**FIGURE 2:**
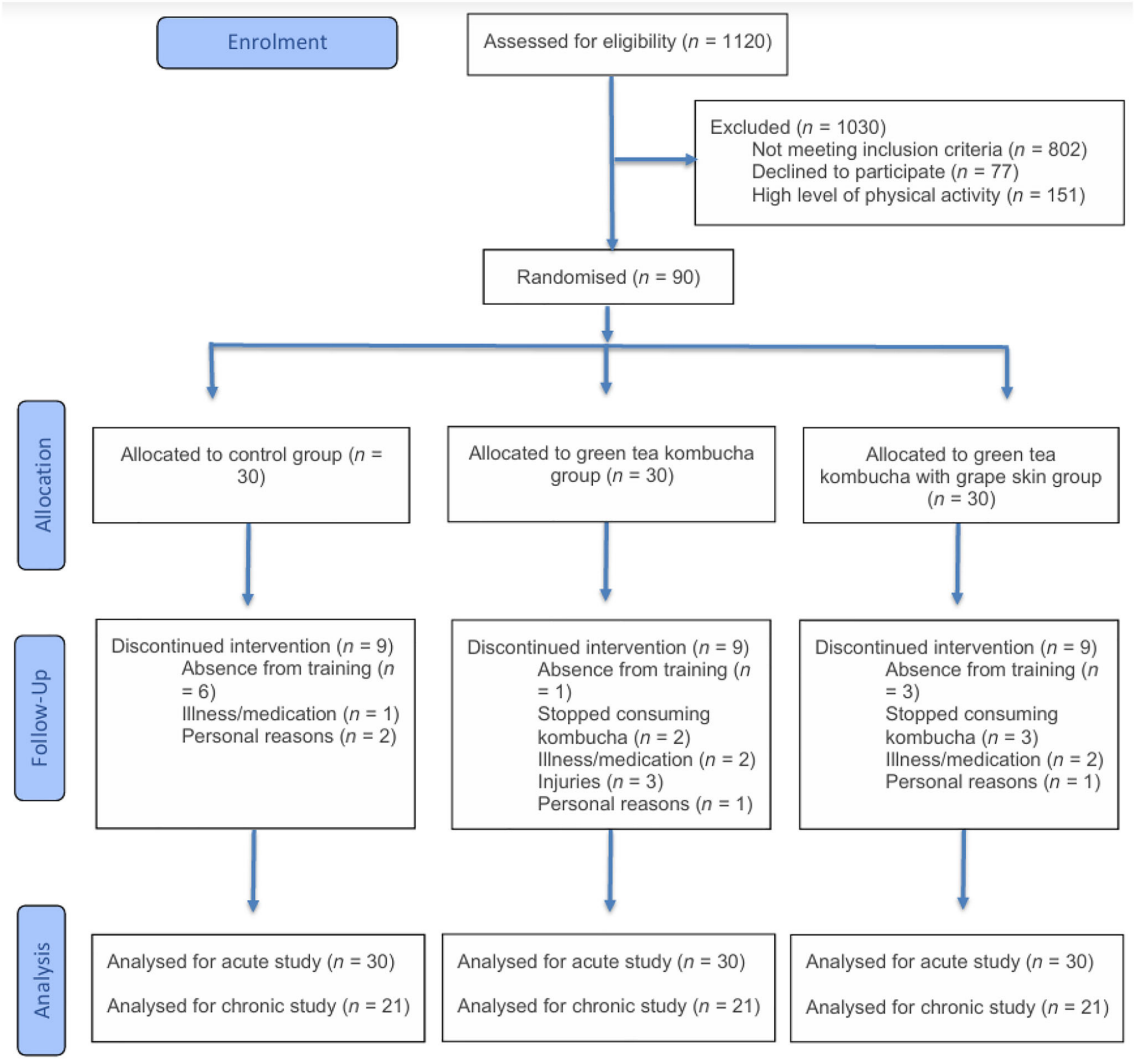
CONSORT 2025 flow diagram.

Consequently, 90 eligible participants were randomized, with 30 allocated to each group, and all completed the acute assessments. During the 12‐week intervention period, 27 participants were discontinued from the study due to poor adherence to the training sessions (*n* = 9), discontinuation of kombucha consumption (*n* = 5), occurrence of diseases and/or use of medications (*n* = 5), musculoskeletal injuries (*n* = 3), or personal reasons (*n* = 5). As a result, 21 participants per group completed the experimental protocol and were included in the chronic assessments, for a total of 63 individuals analyzed (Figure 2). No adverse events were considered to be directly related to kombucha consumption.

Sex distribution did not differ between groups at baseline in either the acute or chronic assessments (acute, *p* = 0.739; chronic, *p* = 0.427). At the acute assessment, the CT‐G and GT‐G groups included 21 women, decreasing to 15 after 12 weeks. The GTG‐G group initially included 18 women, but this number dropped to 10 by study completion. Other baseline characteristics, including age, muscle mass, BMI, total IPAQ score, and fasting glucose, also did not differ among the three groups. Furthermore, baseline characteristics did not differ significantly between participants included in the acute and chronic analyses (Table 2).

**TABLE 2:** Baseline characteristics of participants according to allocation group.

Variables	Group	Acute (*n* = 90)	*p* (group)	Chronic (*n* = 63)	*p* (group)	*p* (acute × chronic)
Age (years)	CT‐G	25.70 (6.86)	0.950	26.00 (6.92)	0.777	0.832
	GTG‐G	25.60 (6.75)		25.30 (6.47)		
	GT‐G	25.20 (6.39)		24.60 (5.96)		
Muscle mass (kg)	CT‐G	27.90 (11.01)	0.864	28.60 (9.37)	0.187	0.881
	GTG‐G	26.90 (5.24)		27.90 (5.37)		
	GT‐G	26.60 (6.07)		25.40 (4.56)		
BMI (kg/m^2^)	CT‐G	22.30 (1.64)	0.435	22.10 (1.75)	0.796	0.986
	GTG‐G	21.80 (1.75)		22.20 (1.64)		
	GT‐G	21.90 (1.90)		21.80 (2.05)		
Total IPAQ (MET)	CT‐G	903.50 (636.38)	0.325	879.90 (510.48)	0.487	0.602
	GTG‐G	1136.50 (689.08)		1099.30 (643.29)		
	GT‐G	1132.30 (748.48)		1015.20 (761.82)		
Blood glucose (mg/dL)	CT‐G	93.80 (7.99)	0.679	92.20 (8.33)	0.505	0.416
	GTG‐G	93.50 (7.69)		92.70 (6.94)		
	GT‐G	94.90 (5.60)		94.50 (5.78)		

*Note*: Values presented as average (standard deviation). Factorial analysis of variance (factorial ANOVA) was used for all variables. CT‐G, control group; GT‐G, green tea kombucha group; GTG‐G, green tea kombucha with grape skin extract group.

Abbreviation: MET: Metabolic equivalent of task.

### 3.2 Food Consumption

Dietary intake did not differ between groups at baseline (*p* > 0.05). It remained unchanged at the end of the intervention, with no significant effects of group, time, or group × time interaction for total energy intake, carbohydrates, protein, or fat (*p* > 0.05) (Table S1).

### 3.3 Immune Markers

In the acute analysis, no main effect of group nor group × time interaction was observed for the immune markers assessed (*p* > 0.05). In contrast, a significant main effect of time (fasting vs. post‐exercise) was identified, characterized by increases in leukocytes, neutrophils, lymphocytes, monocytes, and platelets, along with reductions in eosinophils, MLR, and PLR (*p* < 0.001 for all) (Table 3).

**TABLE 3:** Acute immune changes after kombucha consumption and physical testing.

Variable	Group	Fasting	Post‐exercise	*p* (time)	*p* (group)	*p* (time × group)
Leukocytes (mm3)	CT‐G	5830 (1529)	9010 (2397)	< 0.001*	0.767	0.270
	GTG‐G	6036 (2038)	8339 (2346)			
	GT‐G	5743 (1242)	8439 (2262)			
Neutrophils (%)	CT‐G	53.00 (7.84)	51.96 (11.45)	0.704	0.104	0.697
	GTG‐G	55.31 (8.93)	55.86 (9.26)			
	GT‐G	51.80 (7.57)	52.10 (8.23)			
Neutrophils (mm^3^)	CT‐G	3118 (1027)	4673 (1515)	< 0.001*	0.398	0.885
	GTG‐G	3226 (1105)	4898 (2182)			
	GT‐G	2983 (804)	4437 (1503)			
Eosinophils (%)	CT‐G	2.79 (1.65)	1.82 (1.58)	< 0.001*	0.765	0.911
	GTG‐G	2.72 (2.01)	1.79 (1.67)			
	GT‐G	2.96 (2.20)	2.03 (1.85)			
Eosinophils (mm^3^)	CT‐G	172.43 (131.34)	157.31 (130.91)	0.121	0.536	0.807
	GTG‐G	166.23 (136.13)	127.25 (102.97)			
	GT‐G	197.53 (169.07)	183.42 (202.01)			
Lymphocytes (%)	CT‐G	37.33 (7.97)	40.10 (10.78)	0.005*	0.123	0.670
	GTG‐G	33.93 (10.84)	35.75 (9.44)			
	GT‐G	37.50 (8.26)	39.03 (7.67)			
Lymphocytes (mm^3^)	CT‐G	2161 (727)	3518 (1394)	< 0.001*	0.240	0.410
	GTG‐G	1981 (795)	3012 (1056)			
	GT‐G	2153 (690)	3253 (982)			
Monocytes (%)	CT‐G	6.60 (1.77)	6.00 (1.96)	0.008*	0.231	0.142
	GTG‐G	6.43 (2.24)	6.44 (1.72)			
	GT‐G	7.28 (2.05)	6.75 (1.69)			
Monocytes (mm^3^)	CT‐G	375.07 (120.06)	515.53 (200.01)	< 0.001*	0.699	0.815
	GTG‐G	381.37 (148.91)	549.06 (207.54)			
	GT‐G	408.47 (127.82)	556.21 (167.13)			
Platelets (mm^3^)	CT‐G	242,733 (54,083)	293,379 (64,644)	< 0.001*	0.901	0.719
	GTG‐G	250,066 (62,322)	295,965 (75,290)			
	GT‐G	244,500 (56,780)	290,428 (63,608)			
NLR	CT‐G	1.53 (0.54)	1.49 (0.82)	0.229	0.181	0.970
	GTG‐G	1.77 (0.77)	1.64 (0.70)			
	GT‐G	1.49 (0.52)	1.43 (0.57)			
MLR	CT‐G	0.19 (0.07)	0.16 (0.06)	< 0.001*	0.177	0.525
	GTG‐G	0.21 (0.10)	0.19 (0.07)			
	GT‐G	0.21 (0.09)	0.18 (0.06)			
PLR	CT‐G	121.13 (41.33)	94.52 (42.83)	< 0.001*	0.284	0.893
	GTG‐G	131.66 (45.59)	107.45 (38.70)			
	GT‐G	122.85 (43.18)	96.63 (32.91)			

*Note*: Values presented as average (standard deviation). *Indicates statistical significance. Linear mixed models with a repeated‐measures structure were used for all variables. CT‐G, control group; GT‐G, green tea kombucha group; GTG‐G, green tea kombucha with grape skin extract group.

Abbreviations: MLR, monocyte‐to‐lymphocyte ratio; NLR, neutrophil‐to‐lymphocyte ratio; PLR, platelet‐to‐lymphocyte ratio.

After 12 weeks of intervention, a significant time × treatment interaction was observed for the proportions of fasting neutrophils and lymphocytes. Specifically, the GT‐G group showed a significant increase in neutrophils (95% CI 1.117–11.84, *p* = 0.026) and a decrease in lymphocytes (95% CI −10.33 to −1.177, *p* = 0.023) compared to the other groups. In contrast, the GTG‐G group remained similar to the control group for neutrophils and lymphocytes (*p* > 0.05). For the remaining fasting parameters, a significant main effect of time was observed, independent of group, characterized by increases in eosinophils, monocytes, platelets, MLR, NLR, and PLR counts after the intervention period (*p* < 0.05). Following the post‐exercise period, all groups exhibited increases in eosinophils, monocytes, platelets, MLR, and PLR, along with a reduction in lymphocytes (*p* < 0.05). A significant main effect of group between CT‐G and GTG‐G was observed for MLR (*p* = 0.028), whereas no significant group × time interaction was detected for any marker (*p* > 0.05) (Table 4).

**TABLE 4:** The immune system changes after chronic intervention, depending on the allocation group.

Variables	Group	Initial fasting	Final fasting	*p* (time)	*p* (group)	*p* (time × group)	Initial post‐exercise	Final post‐exercise	*p* (time)	*p* (group)	*p* (time × group)
Leukocytes (mm^3^)	CT‐G	5681 (1589)	5081 (1431)	0.550	0.582	0.055	8661 (2506)	7552 (2279)	0.152	0.902	0.371
	GTG‐G	5585 (1498)	5623 (1567)				8157 (2234)	7671 (2258)			
	GT‐G	5635 (866)	5925 (1380)				7895 (1826)	8022 (2108)			
Neutrophils (%)	CT‐G ^a^	52.7 (8.3)	53.0 (9.6)	0.038*	0.224	0.026*	52.7 (11.8)	52.3 (7.2)	0.217	0.207	0.247
	GTG‐G ^a^	57.3 (9.7)	57.3 (7.6)				55.9 (8.7)	57.7 (9.5)			
	GT‐G ^b^	52.2 (8.31)	59.0 (12.9)				52.9 (8.9)	56.9 (11.4)			
Neutrophils (mm^3^)	CT‐G	3024 (1078)	2743 (1060)	0.531	0.433	0.120	4556 (1562)	3925 (1184)	0.360	0.843	0.073
	GTG‐G	3511 (1979)	3033 (653)				4465 (1328)	4148 (1058)			
	GT‐G	2944 (679)	3354 (1392)				4231 (1447)	4593 (1461)			
Eosinophils (%)	CT‐G	2.5 (1.2)	3.0 (1.5)	0.020*	0.251	0.937	1.5 (0.8)	2.1 (1.3)	0.015*	0.678	0.537
	GTG‐G	3.2 (2.4)	3.8 (2.6)				1.6 (1.3)	2.1 (1.2)			
	GT‐G	2.2 (1.1)	2.8 (2.1)				1.5 (1.3)	1.5 (1.0)			
Eosinophils (mm^3^)	CT‐G	157.8 (110.8)	143.7 (93.9)	0.422	0.301	0.368	125.7 (72.0)	143.5 (75.1)	0.027*	0.482	0.924
	GTG‐G	183.9 (153.3)	213.4 (153.5)				133.0 (106.9)	159.5 (115.2)			
	GT‐G	143.7 (110.9)	159.7 (113.8)				104.6 (89.0)	135.7 (84.3)			
Lymphocytes (%)	CT‐G^a^	37.9 (8.7)	36.3 (8.5)	< 0.001*	0.106	0.023*	39.8 (11.4)	38.5 (7.7)	0.022*	0.080	0.300
	GTG‐G^a^	32.4 (9.0)	30.8 (6.2)				35.2 (8.8)	32.1 (9.6)			
	GT‐G^b^	38.0 (9.6)	30.8 (11.9)				38.6 (8.9)	33.8 (10.4)			
Lymphocytes (mm^3^)	CT‐G	2042 (675)	1808 (589)	< 0.001*	0.511	0.254	3114 (1199)	2906 (1153)	0.073	0.394	0.798
	GTG‐G	1833 (676)	1703 (471)				2921 (940)	2483 (1017)			
	GT‐G	2141 (664)	1776 (658)				3001 (837)	2703 (1096)			
Monocytes (%)	CT‐G	6.5 (1.7)	7.6 (2.1)	0.027*	0.594	0.237	5.5 (1.6)	6.8 (1.6)	0.004*	0.061	0.393
	GTG‐G	6.9 (2.0)	8.0 (1.2)				6.7 (1.7)	7.3 (1.3)			
	GT‐G	7.5 (2.1)	7.4 (1.8)				6.8 (1.6)	7.3 (1.6)			
Monocytes (mm^3^)	CT‐G	361.4 (113.7)	377.7 (109.8)	0.036*	0.212	0.541	474.9 (186.6)	520.3 (197.6)	0.440	0.306	0.625
	GTG‐G	381.0 (139.6)	444.9 (112.0)				565.1 (189.3)	524.2 (194.4)			
	GT‐G	405.1 (142.9)	439.4 (129.0)				537.8 (176.6)	588.0 (204.3)			
Platelets (mm^3^)	CT‐G	240,054 (56,457)	254,090 (48,467)	< 0.001*	0.415	0.415	285,714 (62,116)	290,526 (53,203)	0.014*	0.805	0.325
	GTG‐G	241,047 (62,802)	274,142 (89,918)				284,350 (78,474)	318,476 (98,397)			
	GT‐G	250,030 (59,000)	271,700 (51,289)				292,200 (71,000)	309,111 (76,806)			
NLR	CT‐G	1.52 (0.5)	1.59 (0.6)	0.007*	0.248	0.358	1.55 (0.8)	1.45 (0.5)	0.327	0.556	0.149
	GTG‐G	1.77 (0.6)	1.98 (0.6)				1.57 (0.4)	1.79 (0.7)			
	GT‐G	1.51 (0.5)	1.81 (0.9)				1.50 (0.6)	1.63 (0.6)			
MLR	CT‐G^a^	0.18 (0.1)	0.21 (0.1)	< 0.001*	0.106	0.610	0.15 (0.1)	0.18 (0.1)	0.009*	0.028*	0.664
	GTG‐G^b^	0.21 (0.1)	0.26 (0.1)				0.20 (0.1)	0.22 (0.1)			
	GT‐G^ab^	0.20 (0.1)	0.26 (0.1)				0.18 (0.1)	0.22 (0.1)			
PLR	CT‐G	123.8 (47.1)	150.5 (45.0)	< 0.001*	0.348	0.665	99.1 (46.8)	115.2 (54.2)	< 0.001*	0.544	0.622
	GTG‐G	146.5 (65.4)	169.7 (65.2)				103.9 (35.7)	133.2 (55.3)			
	GT‐G	126.0 (43.5)	164.5 (56.9)				103.7 (34.0)	127.9 (42.4)			

*Note*: Linear mixed models with a repeated‐measures structure were used for all variables. *Indicates statistical significance. Different letters indicate differences between the groups in the post hoc test. CT‐G, control group; GT‐G, green tea kombucha group; GTG‐G, green tea kombucha with grape skin extract group.

Abbreviations: MLR, monocyte‐to‐lymphocyte ratio; NLR, neutrophil‐to‐lymphocyte ratio; PLR, platelet‐to‐lymphocyte ratio.

### 3.4 Oxidative Stress Markers

In the acute intervention, no statistically significant differences were observed between groups for any of the evaluated markers, nor was a significant time × group interaction detected. A significant main effect of time was observed only for CAT activity, which increased significantly over the evaluated period (*p* < 0.001).

After the 12‐week intervention, a significant main effect of group was observed for CAT activity under fasting conditions (*p* = 0.037). Bonferroni‐adjusted post hoc analysis revealed a significant difference between the CT‐G and GT‐G groups (95% CI 2.28 × 10^−^^5^ to 1.98 × 10^−^^4^, *p* = 0.041), with the CT‐G group exhibiting a reduction in CAT activity. In contrast, the GT‐G group showed a small increase in CAT levels following the intervention. For this marker, the GTG‐G group did not significantly differ from the other groups.

A group × time interaction effect was observed for MDA equivalents concentrations in the chronic post‐exercise period (95% CI 0.298–3.212, *p* = 0.033). However, post hoc analysis revealed no significant differences between the treatment groups. Despite the absence of significant between‐group differences in post hoc analyses, the GT‐G group exhibited a marked reduction in MDA equivalents concentrations following the intervention (54.19%).

**FIGURE 3:**
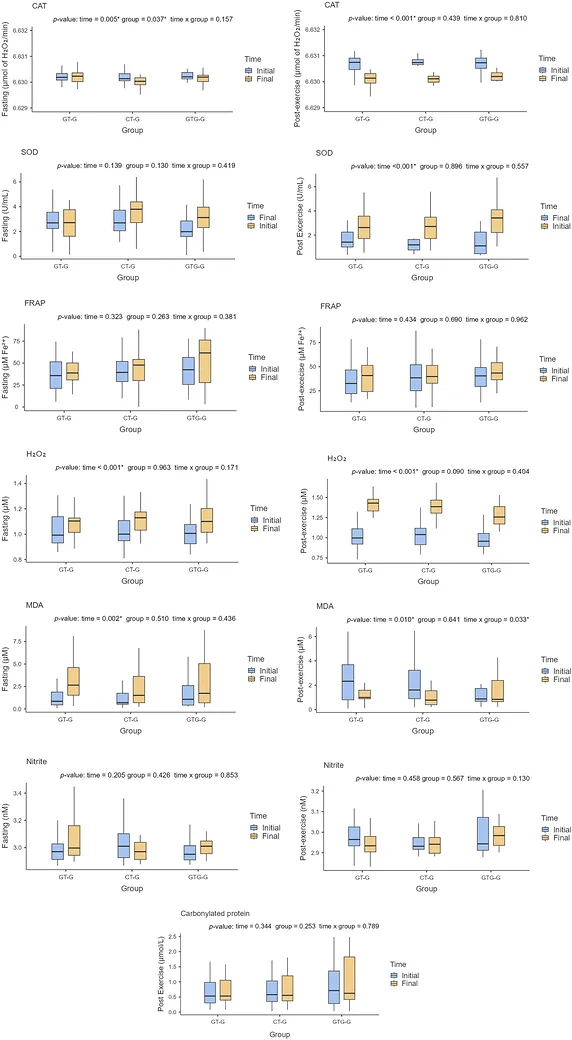
Assessment of oxidative stress markers at baseline and end of the intervention under fasting and post‐exercise conditions according to allocation group. Linear mixed models with a repeated‐measures structure were used for all variables. CT‐G, control group; GT‐G, green tea kombucha group; GTG‐G, green tea kombucha with grape skin extract group. CAT, catalase; FRAP, ferric reducing antioxidant power; H_2_O_2_, hydrogen peroxide; MDA, malondialdehyde equivalents; SOD, superoxide dismutase.

No significant chronic effects of the intervention were observed for other outcomes, either for the main effect of group or for the time × group interaction. Regarding the main effect of time, CAT activity decreased under both fasting and post‐exercise conditions (*p* < 0.05), while SOD activity decreased in the post‐exercise condition (*p* < 0.001). In contrast, MDA equivalents levels increased under fasting conditions (*p* = 0.002) and decreased in the post‐exercise condition (*p* = 0.010). Additionally, H_2_O_2_ levels increased under both fasting and post‐exercise conditions (*p* < 0.001) (Figure 3).

### 3.5 Additional Analyses

**FIGURE 4:**
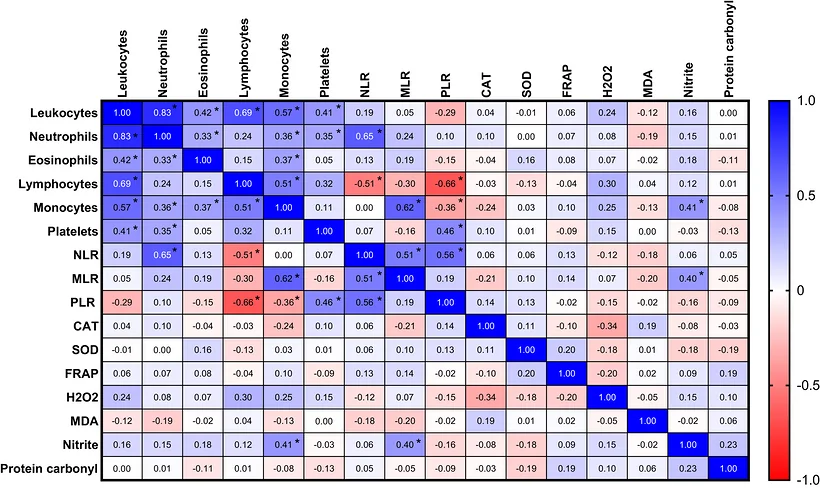
Correlation matrix between immune and oxidative stress markers in the post‐exercise period. ^*^Statistical significance in Spearman's correlation after Benjamini–Hochberg FDR correction. CAT, catalase; FRAP, ferric reducing antioxidant power; H_2_O_2_, hydrogen peroxide; MDA, malondialdehyde equivalents; MLR, monocyte‐to‐lymphocyte ratio; NLR, neutrophil‐to‐lymphocyte ratio; PLR, platelet‐to‐lymphocyte ratio; SOD, superoxide dismutase.

During the post‐exercise period, nitrite levels were moderately and positively correlated with both monocyte count and MLR (*p* = 0.010 and *p* = 0.013, respectively) (Figure 4).

The sensitivity analysis yielded results comparable to those of the primary analysis, with no material changes in the statistical interpretation. The primary outcome, FRAP, remained nonsignificant across both analytical approaches, for both fasting and post‐exercise measurements. The other interactions for the previously identified nominally significant outcomes remained significant when comparing complete‐case and all‐available‐data analyses, supporting the consistency of these findings (Table S2).

After adjustment for multiple comparisons using the Benjamini–Hochberg FDR procedure, none of the interactions identified among the secondary outcomes remained statistically significant at an FDR threshold of 5%. This applied to immune markers assessed under fasting and post‐exercise conditions, as well as oxidative stress markers assessed under fasting and post‐exercise conditions. Therefore, the previously reported nominally significant interactions for neutrophils (%), lymphocytes (%), catalase, and MDA equivalents were considered exploratory findings and did not meet the prespecified criterion for statistical significance after FDR adjustment.

## 4 Discussion

The 12‐week exercise training protocol promoted activation of innate immunity and a low‐grade systemic inflammatory response, as well as increases in oxidative stress markers, including MDA equivalents and H_2_O_2_, while reducing CAT and SOD activity. Kombucha intake did not influence the primary outcome, FRAP, or acute immune or oxidative stress responses to exercise. However, after 12 weeks, green tea kombucha consumption was associated with alterations in the basal immune profile, characterized by modulation of leukocyte distribution. Concomitantly, fasting CAT activity showed a difference between the groups GT‐G and CT‐G. In this context, the GTG‐G group did not differ from the other groups. Additionally, in the chronic post‐exercise period, the GT‐G group exhibited a marked reduction in MDA equivalents concentrations (54.19%), supported by a significant time × treatment interaction, although post hoc analyses did not reveal significant differences between groups.

Acutely, physical exercise induced a transient leukocytosis, characterized by increases in total leukocytes, segmented neutrophils, lymphocytes, monocytes, and platelets, concomitant with a relative reduction in eosinophils and decreases in the MLR and PLR ratios. This pattern reflects a rapid and coordinated immune mobilization involving both innate and adaptive components, consistent with a physiological response to acute physical stress, without clear evidence of immunosuppression or maladaptive inflammatory activation (Allsopp et al. 2023; Emery et al. 2024; Pipitone et al. 2025; Shi et al. 2025; Song et al. 2025). Regarding the chronic effects, after 12 weeks of intervention, a new basal immunological state was established, independent of the experimental group, as evidenced by increased fasting monocyte and platelet counts, along with sustained elevations in indices associated with inflammation, such as the MLR and PLR ratios (Dadouli et al. 2022; Cai et al. 2024; Li et al. 2024; Kösehasanoğulları et al. 2025). In the post‐exercise period, these individuals exhibited greater mobilization of innate immunity, accompanied by a transient reduction in circulating lymphocytes, suggesting an adaptive response to chronic training, with enhanced immune activation and a shift toward a low‐grade inflammatory profile (Campbell and Turner, 2018; Nieman and Wentz, 2019).

In the present study, chronic green tea kombucha consumption was associated with a selective modulation of the immune response over time, reflected by changes in the relative proportions of segmented neutrophils and lymphocytes, resulting in a significant interaction between time and group. The absence of concomitant changes in absolute cell counts suggests that these alterations are primarily driven by leukocyte redistribution and trafficking between the circulation and peripheral tissues, rather than by hematopoietic or pathological inflammatory processes (Peake et al. 2017; Nieman and Wentz, 2019; Simpson et al. 2020). In addition, no changes were observed in indices associated with inflammation. Together, these findings indicate that exercise was the primary driver of the immunological adaptations, whereas green tea kombucha contributed to subtle adjustments in neutrophil and lymphocyte dynamics. The observed effects of green tea kombucha may be attributed to the bioactive compounds present in the beverage, particularly phenolic compounds, which are known to modulate immune cell function and inflammatory signaling pathways, potentially influencing leukocyte trafficking and distribution (Khan and Mukhtar, 2019; Sun et al. 2022).

In relation to the main effect of time, in the acute condition, there was no impairment of redox status at rest, with a tendency toward increased CAT activity, suggesting an initial antioxidant response to exercise. In contrast, after the chronic intervention period, the reduction in fasting CAT activity and post‐exercise SOD activity, concomitant with increased fasting MDA equivalents levels and elevated H_2_O_2_ concentrations under both fasting and post‐exercise conditions, indicates that the training protocol promoted a pro‐oxidant state. At the end of the study, the elevation of H_2_O_2_ both at rest and during the post‐exercise period further supports the persistence of redox imbalance, reflecting a mismatch between reactive oxygen species production and antioxidant defense capacity. This pattern is consistent with repeated exposure to exercise‐induced oxidative stress, in which increased generation of reactive species may exceed endogenous antioxidant capacity (Vargas‐Mendoza et al. 2022; Ruhee and Suzuki, 2024; Çibuk et al. 2025; Sim et al. 2025).

The differences between GT‐G and CT‐G in oxidative stress markers observed after a 12‐week intervention can be explained by the composition of the green tea kombucha. Green tea catechins and other phenolic compounds may modulate redox‐sensitive signaling pathways, particularly the Nrf2 pathway, which regulates the expression of endogenous antioxidant enzymes, such as CAT (Anantachoke et al. 2023; Chou et al. 2024; Fraiz et al. 2025; Li et al. 2025). Enhanced antioxidant defenses may increase the removal of RONS, thereby limiting the initiation and propagation of lipid peroxidation and the subsequent formation of lipid peroxidation‐derived products, including MDA (Jomova et al. 2024; Chandimali et al. 2025). Despite there being no difference between the groups in post hoc tests, the GT‐G group exhibited a marked reduction in post‐exercise MDA equivalents concentrations, suggesting a potential effect of green tea kombucha against exercise‐induced lipid peroxidation (Polli et al. 2019).

Corroborating these findings, nitrite levels were moderately and positively correlated with both monocyte count and MLR. Nitrite is a relatively stable product of nitric oxide metabolism and can also participate in the dynamic interconversion of nitrogen oxides under physiological conditions. Therefore, changes in nitrite concentrations may reflect alterations in the broader nitric oxide/nitrogen oxide pathway, which is closely interconnected with redox and inflammatory processes (Paiva et al. 2024; Liang et al. 2026). Taken together, these findings suggest a possible relationship between preserving redox homeostasis and modulating systemic inflammatory status (Bellanti et al. 2025; Feng et al. 2025).

Interestingly, the possible effects on antioxidant and immune markers were observed only in the GT‐G, with no significant responses detected in the grape‐enriched formulation. Thus, our initial hypothesis that grape skin‐derived phenolics would potentiate the antioxidant effects of kombucha was not supported by the present findings. The biological activity of kombucha is not determined solely by total phenolic content (A. Chen et al. 2025). Despite the higher phenolic concentration in the grape kombucha, differences in the overall chemical profile between the beverages may have contributed to the distinct physiological responses. In particular, the green tea kombucha exhibited higher levels of acetic acid and a lower pH, which may influence phenolic bioavailability and downstream redox signaling (Costa et al. 2023; Cardoso et al. 2020; Cardoso et al. 2021).

Although no significant differences were detected in the outcomes for the grape‐enriched kombucha, in some parameters, GTG‐G exhibited responses comparable to those observed in the GT‐G group. This suggests that the addition of grape skin extract did not attenuate the beverage's biological activity but may have resulted in a distinct modulatory profile. Previous studies have demonstrated that combinations of polyphenols can exert synergistic or additive effects, particularly under conditions of elevated oxidative stress or inflammation (Zhang et al. 2019; X. Chen et al. 2022; Mitra et al. 2023). In this context, the absence of superior effects of GTG‐G in the present study may be related to the relatively healthy and physically active status of the participants (Sarkhosh‐Khorasani et al. 2021; Rudrapal et al. 2022).

This study has several strengths. To the best of our knowledge, this is the first randomized controlled trial to investigate the effects of kombucha consumption in runners, and the first to evaluate a green tea‐based kombucha enriched with grape skin extract on health‐related outcomes. All groups presented similar baseline characteristics and maintained supervised physical activity levels, while dietary intake did not differ among participants throughout the study period.

However, this study has limitations. Although the overall loss to follow‐up was within the anticipated 30% attrition rate and the target sample size was achieved, the unequal distribution of losses across sexes should be acknowledged as a potential source of attrition bias. In addition, participants' awareness of treatment allocation may have influenced their perceptions, self‐reported outcomes, or adherence, potentially introducing reporting or behavioral bias.

FRAP, which was prespecified as the primary outcome of this trial, was not significantly affected by the 12‐week intervention. After adjustment for multiple testing, nominally significant findings for secondary outcomes did not remain significant after Benjamini–Hochberg FDR adjustment and should therefore be interpreted cautiously as exploratory findings.

It must be considered that exercise‐induced changes in plasma volume may have influenced post‐exercise biomarker concentrations. Also, it should be noted that the assessment of the inflammatory response was based on indirect hematological markers, without including circulating cytokines. In addition, menstrual‐cycle phase and hormonal contraceptive use were not specifically controlled in the analyses. We also recognize that the 3‐day food diary may not fully capture habitual dietary antioxidant intake.

As the intervention consisted of a whole kombucha beverage, the study design did not allow the effects of individual kombucha components to be disentangled. Furthermore, the study did not directly assess the bioavailability of its phenolic compounds, limiting the interpretation of their potential contribution to the observed effects. Finally, the study population comprised individuals without prior running experience. The present findings may not be generalizable to trained or experienced runners, who could exhibit distinct physiological responses.

## 5 Conclusions

In conclusion, the 12‐week running training protocol promoted immune adaptation and was associated with a modified oxidative stress profile, characterized by increased fasting MDA equivalents but decreased post‐exercise MDA equivalents, alongside increased H_2_O_2_ and reduced CAT activity under both conditions, as well as reduced post‐exercise SOD activity. The relationship between post‐exercise oxidative stress and immune markers suggests an interaction between antioxidant defense mechanisms and the regulation of inflammation during chronic training adaptations.

In this context, kombucha intake was not associated with significant changes in FRAP or acute exercise responses; however, after 12 weeks, it was associated with changes in leukocyte distribution, fasting catalase activity, and post‐exercise MDA equivalents concentrations. Sensitivity analyses including all randomized participants yielded results consistent with the primary analyses, whereas FDR‐adjusted analyses indicated that these findings should be interpreted as exploratory. Adding grape skin extract did not provide evidence of enhanced antioxidant effects beyond those observed with green tea kombucha alone under the conditions evaluated. Overall, these findings suggest that green tea kombucha may influence immune and oxidative stress markers during chronic running training, although further studies are needed to clarify these findings.

## Author Contributions

**Dandara Baia Bonifácio**: conceptualization, methodology, formal analysis, investigation, writing – original draft, visualization. **Rayanne Santos de Paulo**: methodology, investigation. **Pedro Henrique Viana Mendes**: methodology, investigation. **Udielle Vermelho Lacerda**: methodology, investigation. **Rodrigo Rezende Cardoso**: methodology, investigation. **Helton de Sá Souza**: conceptualization, methodology, resources, writing – review and editing, supervision, project administration, funding acquisition. **Frederico Augusto Ribeiro de Barros**: conceptualization, funding acquisition, methodology, writing – review and editing, project administration, supervision, resources. **Josefina Bressan**: conceptualization, funding acquisition, methodology, writing – review and editing, project administration, supervision, resources.

## Conflicts of Interest

The authors declare no conflicts of interest.

## Supporting information




**Supplementary Material**: jfds71505‐sup‐0001‐Table S1‐S2.docx

## Data Availability

The datasets described in this manuscript, as well as the variable dictionary and analysis code, can be obtained from the corresponding author upon request.
