# Supplementary material for: Acute and Chronic Effects of Green Tea Kombucha With and Without Grape Skin Extract on Immune and Oxidative Stress Markers in Beginner Runners: A Randomized Controlled Trial
Source: J Food Sci. 2026 Sep 26;91(9):e71505. doi: 10.1111/1750-3841.71505 (PMC13615550; doi:10.1111/1750-3841.71505)
Supplement: Supplementary file 1 [file JFDS-91-0-s001.docx]

| Supplementary Table 1. Energy and macronutrient intake by group and time point | | | | | | | |
| --- | --- | --- | --- | --- | --- | --- | --- |
|  | **Group** | **Time** | **Mean** | **SD** | ***p* (time)** | ***p* (group)** | ***p* (time x group)** |
| Energy (Kcal/day) | CT-G | Final | 1987.3 | 727.16 | 0.668 | 0.668 | 0.442 |
|  |  | Initial | 1911.4 | 430.14 |  |  |  |
|  | GT-G | Final | 1739.8 | 569.15 |  |  |  |
|  |  | Initial | 1853.7 | 765.16 |  |  |  |
|  | GTG-G | Final | 1957.4 | 623.49 |  |  |  |
|  |  | Initial | 1796.1 | 577.57 |  |  |  |
| Protein (g) | CT-G | Final | 43.8 | 11.19 | 0.138 | 0.953 | 0.372 |
|  |  | Initial | 44.6 | 9.02 |  |  |  |
|  | GT-G | Final | 45.1 | 7.20 |  |  |  |
|  |  | Initial | 44.7 | 8.09 |  |  |  |
|  | GTG-G | Final | 41.7 | 9.02 |  |  |  |
|  |  | Initial | 47.0 | 12.27 |  |  |  |
| Fat (g) | CT-G | Final | 33.3 | 5.34 | 0.484 | 0.069 | 0.796 |
|  |  | Initial | 34.4 | 8.04 |  |  |  |
|  | GT-G | Final | 36.5 | 5.56 |  |  |  |
|  |  | Initial | 35.9 | 5.97 |  |  |  |
|  | GTG-G | Final | 31.6 | 6.34 |  |  |  |
|  |  | Initial | 32.3 | 7.11 |  |  |  |
| Carbohydrate (g) | CT-G | Final | 132.0 | 19.28 | 0.183 | 0.244 | 0.510 |
|  |  | Initial | 129.6 | 25.40 |  |  |  |
|  | GT-G | Final | 124.4 | 17.35 |  |  |  |
|  |  | Initial | 125.5 | 18.29 |  |  |  |
|  | GTG-G | Final | 139.2 | 21.21 |  |  |  |
|  |  | Initial | 131.7 | 19.56 |  |  |  |

 Macronutrient intake was adjusted per 1,000 kcal. Linear mixed models with a repeated-measures structure were used for all variables. g: grams; kcal/day: kilocalories per day; SD: standard deviation.

**Supplementary Table 2.** Sensitivity analysis of the primary and nominally significant outcomes using complete-case and all-available-data approaches

| Variables | n = 90 | | | n = 63 | | |
| --- | --- | --- | --- | --- | --- | --- |
|  | ***p* (time)** | ***p* (group)** | ***p* (time x group)** | ***p* (time)** | ***p* (group)** | ***p* (time x group)** |
| Neutrophils fasting (%) | 0.022* | 0.285 | 0.017* | 0.038* | 0.224 | 0.026* |
| Lymphocytes fasting (%) | <0.001* | 0.221 | 0.027* | <0.001* | 0.106 | 0.023* |
| FRAP fasting (µM Fe²⁺) | 0.173 | 0.275 | 0.323 | 0.323 | 0.263 | 0.381 |
| FRAP post-exercise (µM Fe²⁺) | 0.426 | 0.828 | 0.935 | 0.434 | 0.690 | 0.962 |
| CAT fasting (µmol of H₂O₂/min) | 0.009* | 0.046* | 0.756 | 0.005* | 0.037* | 0.157 |
| MDA post-exercise (µM) | 0.177 | 0.812 | 0.036* | 0.010* | 0.641 | 0.033* |

Linear mixed models with a repeated-measures structure were used for all variables. CAT: Catalase; FRAP: Ferric Reducing Antioxidant Power; MDA: Malondialdehyde equivalents; µM: micromolar; µM Fe²⁺: micromolar Fe²⁺ equivalents; µmol of H₂O₂/min: micromoles of hydrogen peroxide per minute.
